# Early fulminant BK polyomavirus-associated nephropathy in two kidney transplant patients with low neutralizing antibody titers receiving allografts from the same donor

**DOI:** 10.1186/s12985-019-1275-9

**Published:** 2020-01-10

**Authors:** Elias Myrvoll Lorentzen, Stian Henriksen, Amandeep Kaur, Grete Birkeland Kro, Clara Hammarström, Hans H. Hirsch, Karsten Midtvedt, Christine Hanssen Rinaldo

**Affiliations:** 1 Department of Microbiology and Infection Control, University Hospital of North Norway, Tromsø, Norway; 2 Metabolic and Renal Research Group, UiT The Arctic University of Norway, Tromsø, Norway; 3 Department Biomedicine Transplantation & Clinical Virology, University of Basel, Basel, Switzerland; 4 Department of Microbiology, Oslo University Hospital, Rikshospitalet, Oslo, Norway; 5 Institute of Clinical Medicine, University of Oslo, Oslo, Norway; 6 Department of Pathology, Oslo University Hospital, Rikshospitalet, Oslo, Norway; 7 Infectious Diseases & Hospital Epidemiology, University Hospital Basel, Basel, Switzerland; 8 Department of Transplantation, Medicine, Section of Nephrology, Oslo University Hospital, Rikshospitalet, Oslo, Norway

**Keywords:** Kidney transplantation, BK polyomavirus, BKPyV-DNAemia, PyVAN, Neutralizing antibodies

## Abstract

**Background:**

BK Polyomavirus (BKPyV) causes premature graft failure in 1 to 15% of kidney transplant (KT) recipients. High-level BKPyV-viruria and BKPyV-DNAemia precede polyomavirus-associated nephropathy (PyVAN), and guide clinical management decisions. In most cases, BKPyV appears to come from the donor kidney, but data from biopsy-proven PyVAN cases are lacking. Here, we report the early fulminant course of biopsy-proven PyVAN in two male KT recipients in their sixties, receiving kidneys from the same deceased male donor.

**Case presentations:**

Both recipients received intravenous basiliximab induction, and maintenance therapy consisting of tacrolimus (trough levels 3–7 ng/mL from time of engraftment), mycophenolate mofetil 750 mg bid, and prednisolone. At 4 weeks post-transplant, renal function was satisfactory with serum creatinine concentrations of 106 and 72 μmol/L in recipient #1 and recipient #2, respectively. Plasma BKPyV-DNAemia was first investigated at 5 and 8 weeks post-transplant being 8.58 × 10^4^ and 1.12 × 10^6^ copies/mL in recipient #1 and recipient #2, respectively. Renal function declined and biopsy-proven PyVAN was diagnosed in both recipients at 12 weeks post-transplant. Mycophenolate mofetil levels were reduced from 750 mg to 250 mg bid while tacrolimus levels were kept below 5 ng/mL. Recipient #2 cleared BKPyV-DNAemia at 5.5 months post-transplant, while recipient #1 had persistent BKPyV-DNAemia of 1.07 × 10^5^ copies/mL at the last follow-up 52 weeks post-transplant. DNA sequencing of viral DNA from early plasma samples revealed apparently identical viruses in both recipients, belonging to genotype Ib-2 with archetype non-coding control region. Retrospective serological work-up, demonstrated that the donor had high BKPyV-IgG-virus-like particle ELISA activity and a high BKPyV-genotype I neutralizing antibody titer, whereas both KT recipients only had low neutralizing antibody titers pre-transplantation. By 20 weeks post-transplant, the neutralizing antibody titer had increased by > 1000-fold in both recipients, but only recipient #2 cleared BKPyV-DNAemia.

**Conclusions:**

Low titers of genotype-specific neutralizing antibodies in recipients pre-transplant, may identify patients at high risk for early fulminant donor-derived BKPyV-DNAemia and PyVAN, but development of high neutralizing antibody titers may not be sufficient for clearance.

## Background

BK Polyomavirus (BKPyV) infects about 90% of the world’s population [3, 14]. After primary infection, which usually goes unnoticed, the virus persists quietly in the epithelial cells of the reno-urinary tract. Asymptomatic low-level virus shedding in the urine has been detected in healthy immunocompetent blood donors indicating immune escape of BKPyV [6, 17]. In kidney transplant (KT) recipients, where the immune system is suppressed by immunosuppressive drugs in order to avoid rejection, the prevalence of viruria increases to more than 60%, and about half of these viruric patients develop high-level BKPyV viruria defined as > 7 log_10_ copies (c) per mL and shed decoy cells. About 2 to 6 weeks later, approximately half of these patients progress to BKPyV-DNAemia and biopsy-proven polyomavirus-associated nephropathy (PyVAN). The disease is characterized by persisting high-level BKPyV replication in the tubular epithelial cells of the kidney allograft, causing cytopathic loss. The disruption of the epithelial cell monolayer leads to leakage of virus and viral DNA into the tissue and blood stream i.e. BKPyV DNAemia, and is followed by a local inflammation [4, 12, 22]. In addition, high-level BKPyV replication in the multilayered epithelium of the renal pelvis and the bladder, contribute to the viruria. As antiviral drugs for treatment of PyVAN are lacking, the mainstay therapy is a stepwise reduction of immunosuppression [13]. Without this intervention, more than 90% of affected KT recipients will show a declining kidney allograft function and experience premature graft loss.

BKPyV has a circular double-stranded DNA genome of about 5 kb. The genetic heterogeneity in the *VP1* gene encoding the major capsid protein Vp1, can be used to divide BKPyV into four sero−/genotypes (I, II, III, IV) [15], two of which can be further divided into subtypes (Ia, Ib-1, Ib-2, Ic, IVa-I, IVa-2, IVb-1, IVb-2, IVc-I and IVc-2) [38]. Another genome sequence used to characterize the virus is the non-coding control region (*NCCR*) which comprises the origin of viral genome replication and promoter/enhancer functions. In urine from immunocompetent individuals, BKPyV typically has an archetype NCCR architecture that has been arbitrarily divided into five sequence blocks denoted O_142_ - P_68_ - Q_39_ - R_63_ - S_63_, where the subscript number indicates the number of base pairs. Early in the course of PyVAN, BKPyV strains with an archetype NCCR are found in urine and plasma. Presumably due to the lack of a functional T-cell immunity, these strains are gradually replaced by faster replicating strains with a rearranged NCCRs showing an upregulated expression of the early regulatory protein large T-antigen (LTag) [9, 23, 24].

Since PyVAN preferentially affects KT recipients, PyVAN has been suggested to arise mainly due to donor-derived infection [2]. This concept is supported by the detection of identical BKPyV-genotypes and/or strains in the donor urine pre-transplant and in the recipients urine and/or plasma post-transplant [2, 29, 30, 35, 37]. Moreover, a study of 21,575 recipient pairs receiving kidneys from the same donor supported this concept, as BKPyV replication was reported in twice as many recipient pairs (*n* = 174) than expected by chance [32]. However, data from recipient pairs with biopsy-proven nephropathy are lacking.

Here, we describe the course of two KT patients developing early fulminant biopsy-proven PyVAN after receiving their allografts from the same deceased donor. Retrospective sequencing of the BKPyV genome indicated that PyVAN developed as a result of transmission of donor-derived BKPyV. Detailed serological studies identified low neutralizing antibody titers in both recipients pre-transplant as a potential marker of low antiviral immune control and increased risk for BKPyV-DNAemia and PyVAN. Although both recipients developed a more than 1000-fold increase in neutralizing antibody (NAb) titers, only one recipient cleared BKPyV-DNAemia. The potential role of viral and immune markers for screening, monitoring and follow-up is discussed.

## Case presentation

### Deceased donor

The donor was a 62-year old male who died from a subarachnoid hemorrhage. He was IgG-seropositive for cytomegalovirus (CMV) and had blood group A. Retrospective investigation of his plasma using three different serological methods (reviewed in [17]) demonstrated high-levels of BKPyV neutralizing antibodies. In more detail, using a neutralization assay, a more than 50% inhibition of genotype I-pseudovirus infectivity was obtained when a 640-fold plasma dilution was used, which corresponds to a NAb titer of 640 half maximal inhibitory concentration (IC_50_). The method used was modified from a protocol by Pastrana and colleagues [25] by using a pseudovirus containing pEGFP-N1 instead of phGluc. As a consequence, infectivity was measured as fluorescent intensity instead of luciferase activity. The hemagglutination inhibition assay (HIA) [21], measured a HIA-titer of 320. Finally, the BKPyV-IgG specific enzyme-linked immunosorbent assay (ELISA) using Vp1-derived virus-like particles [16], gave a normalized optical density (nOD) of 2.329 for a plasma dilution of 400, but no IgM was detectable. Moreover, using a validated quantitative real-time PCR assay [5], no BKPyV-DNA was detectable in the donor plasma. Besides, immunohistochemistry of the baseline kidney biopsy using a commercial antibody directed against SV40 LTag (Pab416, Merck) but known to cross-react with BKPyV LTag, was negative.

### Case 1

**Fig. 1:**
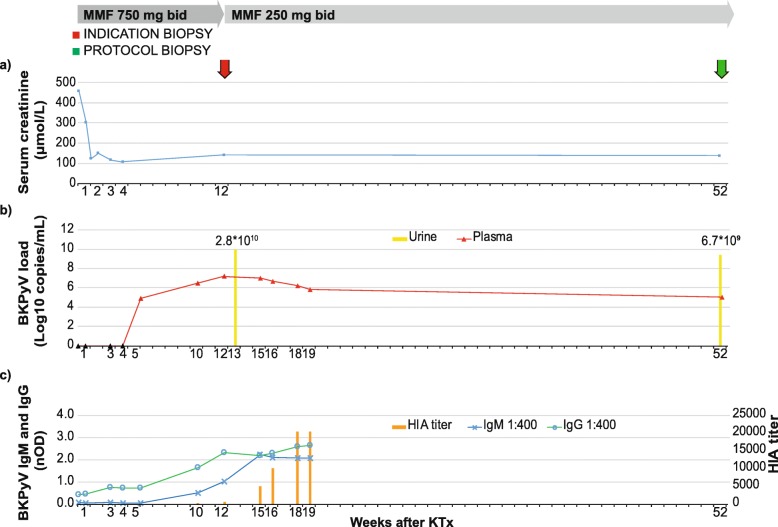
Treatment and Clinical Course of Recipient #1. The X-axis is labeled with the week after transplantation when the sample was taken. Arrows indicate biopsies. Y-axis: **a** Serum creatinine concentration in μmol/L. **b** BKPyV-DNA genome levels in plasma (red triangles) and in urine (yellow bar) in log_10_ c/mL. Retrospectively tested plasma samples (black triangles). **c** BKPyV-antibody IgG (green line) and IgM (blue line) shown as nOD at the left Y-axis; HIA-titer (orange bar) at the right Y-axis

Recipient #1 was a 68 year old male with end-stage kidney disease due to granulomatosis with polyangiitis requiring hemodialysis for the last two years. At the time of transplantation, he had a serum creatinine (s-Cr) of 457 μmol/L (Fig. 1a). Human leukocyte antigen (HLA) typing showed one HLA-A, one HLA-B and one HLA-DR mismatches. His blood group was the same as for the donor and he was seropositive for CMV-IgG, thus yielding an intermediate risk for CMV (D+/R+). No known panel reactive antibody (PRA) or donor specific antibodies (DSA) were detected i.e. the recipient had a standard immunologic risk. He received standard immunosuppressive therapy; intravenous (i.v.) basiliximab induction, prednisolone, tacrolimus (trough levels 3–7 ng/mL from time of engraftment), and mycophenolate mofetil (MMF) 750 mg bid. Four days post-transplant, his s-Cr level was 302 μmol/L, decreasing to 106 μmol/L by 4 weeks post-transplant (Fig. 1a). One week later (5 weeks post-transplant), his plasma was, for the first time, analyzed for BKPyV-DNAemia and 8.58 × 10^4^ c/mL were detected (Fig. 1b), giving him the diagnosis presumptive PyVAN.

**Fig. 2:**
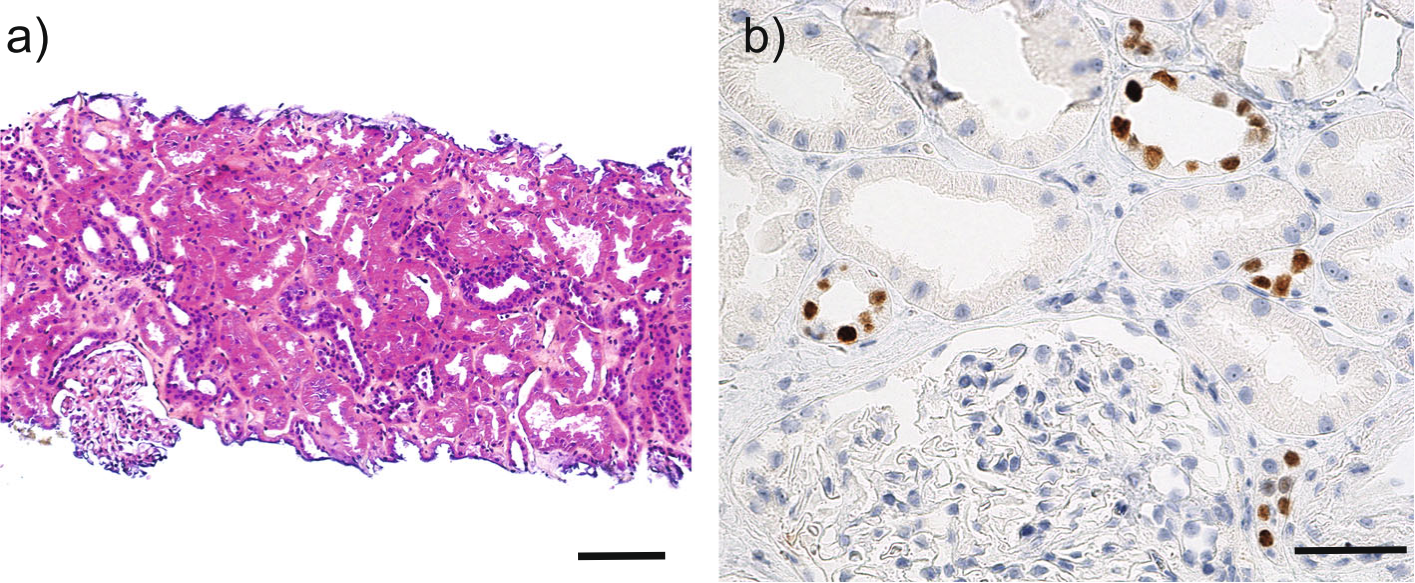
Histological analysis of a renal allograft biopsy from recipient #1 at 12 weeks post-transplant. **a** HES (hematoxylin, eosin and saffron) stained section. Original magnification 200x, scale bar =100 μm. **b** Immunohistochemistry staining of the same biopsy as in a), viral LTag expression (brown colour) in tubular epithelial cells using the cross-reacting monoclonal anti-SV40 LTag antibody Pab416 (Merck). Original magnification 400x, scale bar =50 μm

At 12 weeks post-transplant, his BKPyV plasma load had increased by 3 orders of magnitude to 1.66 × 10^7^ c/mL (Fig. 1b), and the s-Cr level had increased to 139 μmol/L (Fig. 1a). Therefore, an allograft biopsy was taken. The biopsy showed no interstitial inflammation, no intimal arteritis, and no rejection, but mild tubulitis (Banff score of i0t1v0, C4d negative) (Fig. 2a). In addition, positive immunostaining for LTag was observed in some tubular epithelial cells (Fig. 2b), establishing the diagnosis of proven-PyVAN (Stage-B1) [13]. Therefore, MMF was reduced from 750 mg to 250 mg bid while tacrolimus treatment with already low trough levels was left unchanged.

Seven weeks later (19 weeks post-transplant), the plasma BKPyV load had decreased to 6.35 × 10^5^ c/mL (Fig. 1b). Subsequently, the patient was seen in his local hospital, where the s-Cr was reported as stable and plasma BKPyV-DNAemia was not examined. At the planned one-year post-transplant surveillance control, the s-Cr was stable at 135 μmol/L, the plasma BKPyV-load was still 1.07 × 10^5^ c/mL (Fig. 1b), and the urine BKPyV-load was high with 6.71 × 10^9^ c/mL (Fig. 1b). The protocol biopsy showed no signs of inflammation or rejection (Banff score of i0t0v0, C4d negative) and no detectable LTag staining (results not shown) (Fig. 1a).

Retrospective testing of plasma samples taken the first four weeks post-transplant did not detect BKPyV-DNAemia (Fig. 1b, black triangles). Nevertheless, BKPyV-ELISA revealed that recipient #1 was IgG seropositive (0.442 nOD) and IgM seronegative pre-transplantation. Of note, the pre-transplant HIA-titer was 80 (Fig. 1c), and the BKPyV-genotype I NAb-titer was only 10 IC_50._

During the first 5 weeks post-transplant, a slow but continuous increase of the ELISA-IgG activity was found. Then a more rapid increase was seen with a peak value of nOD 2.646 at 19 weeks post-transplant (the last measured time point). During this last phase, the BKPyV-IgM became detectable and peaked at 15 weeks post-transplant (Fig. 1c), indicating a significant immune response to BKPyV-antigens.

At 19 weeks post-transplant, the ELISA IgG and the HIA-titer had increased by six-fold and 256-fold, whereas the NAb-titer had increased by > 1000-fold to > 10,240 IC_90_ i.e. the plasma inhibited more than 90% of the infectious activity at 1:10240 dilution.

### Case 2

**Fig. 3:**
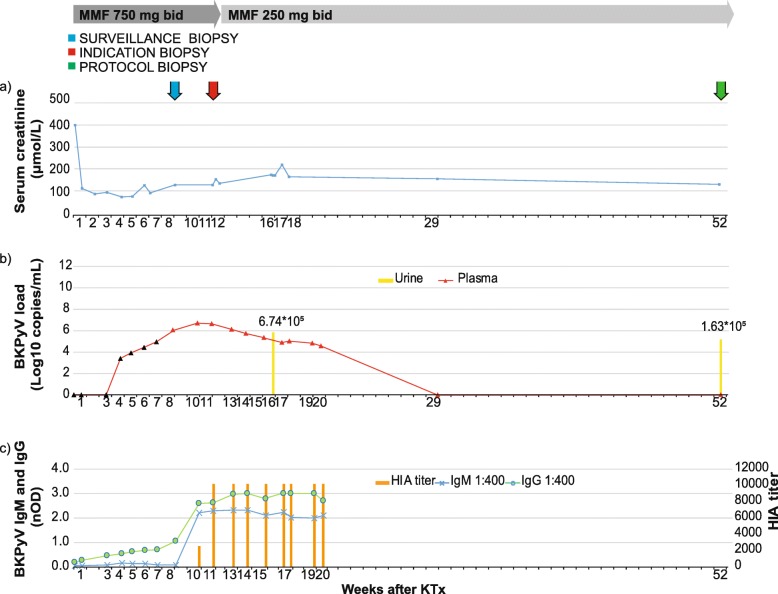
Treatment and Clinical Course of Recipient #2. The X-axis is labeled with the week after transplantation when the sample was taken. Arrows indicate biopsies. Y-axis: **a** Serum creatinine concentration in μmol/L. **b** BKPyV-DNA genome levels in plasma (red triangles) and in urine (yellow bar) in log_10_ c/mL. Retrospectively tested plasma samples (black triangles). **c** BKPyV-antibody IgG (green line) and IgM (blue line) shown as nOD at the left Y-axis; HIA-titer (orange bar) at the right Y-axis.

Recipient #2 was a 62 year old male with autosomal polycystic kidney disease. He had a s-Cr of 401 μmol/L pre-transplantation (Fig. 3a). HLA typing showed one HLA-A, two HLA-B, and one HLA-DR mismatches. The recipient’s blood group was the same as the donor’s and he had an intermediate risk for CMV (D+/R+). No known PRA or DSA were detected and he received the same immunosuppressive therapy as recipient #1. His baseline renal function was good with serum s-Cr levels decreasing from 112 μmol/L at 4 days post-transplant to 72 μmol/L at 5 weeks post-transplant (Fig. 3a).

**Fig. 4:**
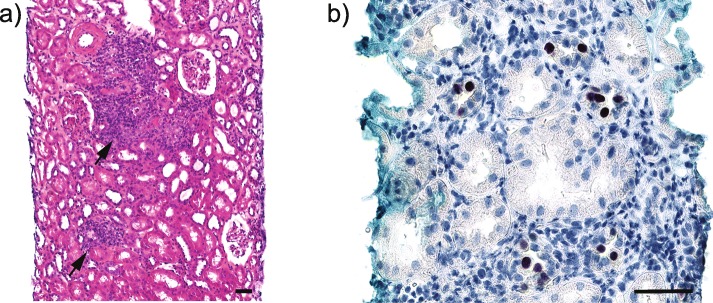
Histological analysis of a renal allograft biopsy from recipient #2 at 12 weeks post-transplant. **a** HES (hematoxylin, eosin and saffron) stained section showing inflammation (arrows). Original magnification 200x, scale bar = 100 μm. **b** Immunohistochemistry staining of the same biopsy as in a), showing LTag expression (brown colour) in tubular epithelial cells when the monoclonal anti–SV40 LTag antibody Pab416 (Merck) is used. Original magnification 400x, scale bar = 50 μm

However, at 6 weeks post-transplant, the s-Cr suddenly increased to 124 μmol/L (Fig. 3a). At 8 weeks post-transplant, the plasma was for the first time analyzed for BKPyV-DNAemia and 1.12 × 10^6^ c/mL was detected (Fig. 3b), giving the diagnosis of presumptive PyVAN. An allograft biopsy was taken, but HES staining showed no signs of inflammation or rejection (Banff score i0t0v0, C4d negative) and immunohistochemical staining was negative for LTag (data not shown). The plasma BKPyV-DNAemia persisted at levels > 6 log_10_ c/mL (Fig. 3b), and at 12 weeks post-transplant a second allograft biopsy was taken. This time the biopsy showed focal interstitial inflammation and severe tubulitis (Banff score i2t3v0, C4d negative) (Fig. 4a). In addition, immunostaining revealed LTag-positive epithelial cells (Fig. 4b) giving the diagnosis of biopsy-proven PyVAN (stage B1). MMF was reduced from 750 mg to 250 mg bid, while tacrolimus treatment was left unchanged (trough levels ng/mL). At 20 weeks post-transplant, the plasma BKPyV-DNA load had declined to 3.56 × 10^4^ c/mL and at 29 weeks post-transplant, BKPyV-DNAemia was no longer detectable (Fig. 3b). Concurrently the s-Cr was 155 μmol/L (Fig. 3a). One year post-transplantation, the s-Cr had declined to 130 μmol/L (Fig. 3a), plasma was still negative for BKPyV-DNAemia (Fig. 3b) while urine was positive with a low BKPyV load of 1.6 × 10^5^ c/mL (Fig. 3b). The protocol biopsy showed limited inflammation and mild tubulitis (Banff score of i1t1v0, corresponding to Banff borderline for rejection, C4d negative) and negative LTag staining (results not shown).

Retrospective testing of plasma BKPyV-DNAemia revealed 2.59 × 10^3^ c/mL in plasma already at 4 weeks post-transplant (Fig. 3b, black triangles). Besides, BKPyV-ELISA demonstrated that recipient #2 was IgG seropositive (nOD of 0.191) and IgM seronegative pre-transplantation. As for recipient #1, the pre-transplant HIA-titer was 80 (Fig. 3c), and the BKPyV-genotype I NAb-titer was only 10 IC_50_. During the first 7 weeks post-transplant, a slow but continuous increase of the BKPyV-IgG titer was found. Then a more rapid increase was seen until the IgG titer plateaued from 13 weeks post-transplant with a maximum nOD of 3.017 at 17 weeks post-transplantation. From 4 weeks post-transplant the BKPyV-IgM became positive and from 11 weeks post-transplant the HIA-titer peaked with 10,240 (Fig. 3c). At 20 weeks post-transplant, the ELISA IgG and the HIA-titer had increased by 16-fold and 128-fold, whereas the NAb-titer had increased by > 1000-fold to > 10,240 IC_90._

### Genetic analysis of BKPyV DNA in plasma and urine samples from both patients

In order to investigate the genotype and strain of BKPyV in plasma and urine samples, two nested PCRs were used to amplify a 330 base pair fragment of the VP1 gene and the complete NCCR [19]. The sequence results from both early plasma samples and urine samples from both recipients revealed virus of genotype Ib-2 having identical archetype NCCR. These results suggest that both recipients were infected with an identical BKPyV strain. However, one year post-transplant plasma sample of recipient #1, also contained strains with NCCR rearrangements, including one strain denoted RH-20 (GenBank Accession number MN627732), having a 60 bp deletion in the Q- and R-block removing the Sp1–4 transcription factor binding site [1].

## Discussion and conclusions

In this study we report the parallel onset of early fulminant biopsy-proven PyVAN in two KT patients having received one kidney each from the same deceased donor. DNA sequencing of BKPyV DNA amplified from early plasma and urine samples, revealed an apparently identical virus of genotype Ib-2 with archetype NCCRs, in both recipients. This together with the clinical course, supports the notion of donor kidney transmission of BKPyV. Both recipients shared several previously reported risk factors for PyVAN [13] such as being males in their sixties and receiving treatment with tacrolimus-mycophenolic acid, whereas other risk factors such as lymphocyte-depleting induction or acute rejection episodes treated with steroid pulses were not present.

Our retrospective analyses revealed that the donor and both recipients were BKPyV-IgG seropositive before transplantation, but significantly differed in their NAb-titers for the replicating BKPyV genotype, which was almost 100-fold higher in the donor than in the recipients. These observations in the recipients are in line with a recent study by Solis and colleagues [31]. They reported that low NAb-titers against the donor BKPyV genotype, here defined as less than 4 log_10_ IC_50_, was associated with an increased risk of BKPyV-DNAemia and PyVAN. Despite this striking similarity, the titers may not be directly comparable, since they used a slightly different protocol.

Remarkably, the BKPyV-genotype I NAb-titers increased in both of our patients by more than 1000-fold to 10,240 IC_90_, thereby, reaching titers associated with clearance of BKPyV DNAemia [31]. Indeed, following MMF reduction, BKPyV-DNAemia declined in recipient #2, and cleared with 3 months. In contrast, recipient #1 had persistent BKPyV-DNAemia levels above 10^5^ c/mL and high-level viruria detectable at one year post-transplant. Moreover, as previously reported [9, 23], the archetype NCCR of the BKPyV genome was now replaced by a rearranged NCCR in line with on-going intra-patient evolution and insufficient antiviral immunity. In particular, CD8 T cells directed against immunodominant 9mer epitopes derived from the viral early protein LTag has been implicated in clearance of BKPyV-DNAemia [17, 20]. Such immunodominant epitopes are presented by HLA-B51 which alone or in combination with HLA-B7 and -B8 has been associated with a lower risk of BKPyV DNAemia [34, 36]. Both recipients lacked these HLA types, except recipient #2 having HLA-B7. Possibly, lack of these HLA-types contributed to the rapid onset and protracted course of PyVAN.

Although we cannot exclude a synergizing role of neutralizing antibodies in the control of BKPyV replication in the affected tubulus of a given nephron, it remains unclear how sufficient antibodies can prevent the well documented cell to cell spread in the nephron.

We noted that the donor was in an age group that is characterized by low titers of BKPyV-specific IgG [10, 18, 28]. In our comprehensive serological assessment using three different assays, however, the donor had high BKPyV-IgG ELISA activity (2.329 nOD), a high HIA-titer (320) as well as a high NAb-titer (> 640 IC_50_). These results suggest that the immune system of the donor had been exposed to BKPyV recently. Considering the donor’s age and the undetectable BKPyV-IgM, this exposure was probably not due to a primary infection, but rather a recent reactivation leading to increased viral loads in his kidneys. Although no pre-transplant viruria samples from the donor were available, the high neutralizing activity against BKPyV of genotype I and the fact that BKPyV genotypes are serologically distinct [26], argues for transmission of genotype I, which also was found in the recipients.

Our parallel kidney transplant case studies from a single donor are also notable for further specific details. Unlike in the donor, the BKPyV-specific antibodies measured by ELISA and by the neutralization assay were discordant in both recipients with respect to the level at the time of transplantation, being higher in the former assay, but nearly undetectable in the latter. This suggest that the ELISA is more sensitive, but less specific for a given BKPyV genotype than the neutralization assay. This may also explain the lack of association of recipient ELISA antibody levels with BKPyV-DNAemia seen in a recent study of living donor-recipient pairs [11]. Moreover, from three weeks post-transplant, the ELISA titers started to increase suggesting a CD4-T cell help independent memory B-cell response to viral antigen exposure, for example resulting from donor virus replication in both kidney allografts directly after transplantation. This interpretation is supported by the fact that the antibody levels increased in parallel with increasing BKPyV-DNAemia before immunosuppression was reduced.

Another aspect is the observation that the first biopsy of recipient #2 was negative for BKPyV-LTag expression although BKPyV-DNAemia was higher than > 10^6^ c/ml. Only a second biopsy taken 4 weeks later confirmed proven PyVAN. This suggests that the biopsy must have missed the typically focally arranged LTag positive epithelial cells, which has been previously documented in a study involving 41 KT recipients with persisting high-level BKPyV-DNAemia [4]. In this study multiple biopsy cores were taken at the same time, and discordant LTag-positive and LTag-negative biopsy cores were found in more than 30% of the cases. The focal nature of PyVAN may also explain why the baseline biopsy at transplantation and the protocol biopsy taken one year post-transplant of recipient #1 were negative. Cases of allograft nephrectomy have clearly demonstrated that BKPyV-DNAemia is derived directly from the renal allograft [7, 8] and BKPyV-DNAemia is now considered a direct biological marker of PyVAN [13]. Importantly this has been implemented in the recently updated guidelines on BKPyV in solid organ transplantation [13]. A renal allograft biopsy is only needed to decide on immunosuppression reduction in patients with an increased risk of acute rejection (i.e. the presence of DSA or known PRA positivity) or impaired baseline renal function of unknown origin. For all other patients, a preemptive treatment algorithm is recommended. To better reflect the continuum of BKPyV replication, immunosuppression reduction is recommended for KT patients with plasma BKPyV-DNAemia of 1000 c/ml sustained for more than three weeks (probable PyVAN), or more than 10,000 c/ml (presumptive PyVAN).

Finally, while supporting the potential of neutralizing antibodies as markers of increased risk, our case studies raise questions about the potential of neutralizing antibodies for prophylaxis or therapy. As commercial human i.v. immunoglobulin (Ig) has been shown to contain BKPyV neutralizing antibodies [27], recently monthly i.v. Ig injections during the first three critical months post-transplant was suggested as an initiative to prevent PyVAN development [33]. Others have suggested pre-vaccination of KT recipients with a multivalent VLP-based vaccine against all BKPyV sero−/genotypes [25]. However, the question has been raised whether or not the apparently beneficial neutralizing antibody activity observed in patients represents surrogates of their corresponding CD4 and/or CD8 activity (reviewed in [17, 20]). It is conceivable that the efficacy of administrating intravenous immunoglobulins may differ when given prophylactically before significant BKPyV spread in the renal allograft has occurred, or when administered in patients with significant BKPyV-DNAemia and PyVAN. Randomized controlled clinical trials are needed to address both situations. However, our study and that of others suggests that the antibody status pre-transplantation should be assessed in order to obtain meaningful results.

In this paired kidney case report, donor-derived transmission with rapid progression to presumptive and proven PyVAN probably occurred due to the combination of a recent BKPyV exposure in the donor and initial low levels of BKPyV-genotype I neutralizing antibodies in both recipients. More evidence is needed to evaluate whether measurement of neutralizing antibodies pre-transplant can be useful in organ allocation or more intense post-transplant screening. Until then, monthly screening for BKPyV-DNAemia followed by a rapid reduction of immunosuppression remains the standard measure to prevent allograft damage and loss due to PyVAN.

## Data Availability

Data sharing is not applicable to this article as no datasets were generated or analyzed during the current study.

## Abbreviations

Bid: Twice a day
BKPyV: BK polyomavirus
c: Copies
CMV: Cytomegalovirus
DSA: Donor specific antibodies
ELISA: Enzyme-linked immunosorbent assay
HIA: Hemagglutination inhibition assay
HLA: Human leukocyte antigen
i.v: Intravenous
IC: Inhibitory concentration
KT: Kidney transplant
LTag: Large tumor antigen
MMF: Mycophenolate mofetil
NAb: Neutralizing antibody
NCCR: Non-coding control region
nOD: Normalized optical density
PRA: Panel reactive antibody
PyVAN: Polyomavirus-associated nephropathy
s-Cr: Serum creatinine

## References

[1] Bethge T, Hachemi HA, Manzetti J, Gosert R, Schaffner W, Hirsch HH (2015). Sp1 sites in the noncoding control region of BK polyomavirus are key regulators of bidirectional viral early and late gene expression. J Virol.

[2] Bohl DL, Storch GA, Ryschkewitsch C, Gaudreault-Keener M, Schnitzler MA, Major EO, Brennan DC (2005). Donor origin of BK virus in renal transplantation and role of HLA C7 in susceptibility to sustained BK viremia. Am J Transplant.

[3] DeCaprio JA, Garcea RL (2013). A cornucopia of human polyomaviruses. Nat Rev Microbiol.

[4] Drachenberg CB, Papadimitriou JC, Hirsch HH, Wali R, Crowder C, Nogueira J, Cangro CB, Mendley S, Mian A, Ramos E (2004). Histological patterns of polyomavirus nephropathy: correlation with graft outcome and viral load. Am J Transplant.

[5] Dumoulin A, Hirsch HH (2011). Reevaluating and optimizing polyomavirus BK and JC real-time PCR assays to detect rare sequence polymorphisms. J Clin Microbiol.

[6] Egli A, Infanti L, Dumoulin A, Buser A, Samaridis J, Stebler C, Gosert R, Hirsch HH (2009). Prevalence of polyomavirus BK and JC infection and replication in 400 healthy blood donors. J Infect Dis.

[7] Funk GA, Gosert R, Comoli P, Ginevri F, Hirsch HH (2008). Polyomavirus BK replication dynamics in vivo and in silico to predict cytopathology and viral clearance in kidney transplants. Am J Transplant.

[8] Funk GA, Steiger J, Hirsch HH (2006). Rapid dynamics of polyomavirus type BK in renal transplant recipients. J Infect Dis.

[9] Gosert R, Rinaldo CH, Funk GA, Egli A, Ramos E, Drachenberg CB, Hirsch HH (2008). Polyomavirus BK with rearranged noncoding control region emerge in vivo in renal transplant patients and increase viral replication and cytopathology. J Exp Med.

[10] Gossai A, Waterboer T, Nelson HH, Doherty JA, Michel A, Willhauck-Fleckenstein M, Farzan SF, Christensen BC, Hoen AG, Perry AE, Pawlita M, Karagas MR (2016). Prospective study of human polyomaviruses and risk of cutaneous squamous cell carcinoma in the United States. Cancer Epidemiol Biomark Prev.

[11] Grellier J, Hirsch HH, Mengelle C, Esposito L, Hebral AL, Belliere J, Weissbach F, Izopet J, Del Bello A, Kamar N (2018). Impact of donor BK polyomavirus replication on recipient infections in living donor transplantation. Transpl Infect Dis.

[12] Hirsch HH (2005). BK virus: opportunity makes a pathogen. Clin Infect Dis.

[13] Hirsch HH, Randhawa PS, AST Infectious Diseases Community of Practice. BK polyomavirus in solid organ transplantation-Guidelines from the American Society of Transplantation Infectious Diseases Community of Practice. Clin Transpl. 2019;33:e13528. 10.1111/ctr.13528 30859620

[14] Hirsch HH, Steiger J (2003). Polyomavirus BK. Lancet Infect Dis.

[15] Jin L, Raptis L (2001). Molecular methods for identification and genotyping of BK virus. SV40 Protocols.

[16] Kardas P, Leboeuf C, Hirsch HH (2015). Optimizing JC and BK polyomavirus IgG testing for seroepidemiology and patient counseling. J Clin Virol.

[17] Kaur Amandeep, Wilhelm Maud, Wilk Sabrina, Hirsch Hans H. (2019). BK polyomavirus-specific antibody and T-cell responses in kidney transplantation. Current Opinion in Infectious Diseases.

[18] Kean JM, Rao S, Wang M, Garcea RL (2009). Seroepidemiology of human polyomaviruses. PLoS Pathog.

[19] Koskenvuo M, Dumoulin A, Lautenschlager I, Auvinen E, Mannonen L, Anttila VJ, Jahnukainen K, Saarinen-Pihkala UM, Hirsch HH (2013). BK polyomavirus-associated hemorrhagic cystitis among pediatric allogeneic bone marrow transplant recipients: treatment response and evidence for nosocomial transmission. J Clin Virol.

[20] Leboeuf C, Wilk S, Achermann R, Binet I, Golshayan D, Hadaya K, Hirzel C, Hoffmann M, Huynh-Do U, Koller MT, Manuel O, Mueller NJ, Mueller TF, Schaub S, van Delden C, Weissbach FH, Hirsch HH, Swiss Transplant Cohort S (2017). BK polyomavirus-specific 9mer CD8 T cell responses correlate with clearance of BK viremia in kidney Transplant recipients: first report from the Swiss Transplant Cohort study. Am J Transplant.

[21] Neel JV, Major EO, Awa AA, Glover T, Burgess A, Traub R, Curfman B, Satoh C (1996). Hypothesis: "rogue cell"-type chromosomal damage in lymphocytes is associated with infection with the JC human polyoma virus and has implications for oncopenesis. Proc Natl Acad Sci U S A.

[22] Nickeleit V, Hirsch HH, Binet IF, Gudat F, Prince O, Dalquen P, Thiel G, Mihatsch MJ (1999). Polyomavirus infection of renal allograft recipients: from latent infection to manifest disease. J Am Soc Nephrol.

[23] Olsen GH, Andresen PA, Hilmarsen HT, Bjorang O, Scott H, Midtvedt K, Rinaldo CH (2006). Genetic variability in BK virus regulatory regions in urine and kidney biopsies from renal-transplant patients. J Med Virol.

[24] Olsen GH, Hirsch HH, Rinaldo CH (2009). Functional analysis of polyomavirus BK non-coding control region quasispecies from kidney transplant recipients. J Med Virol.

[25] Pastrana DV, Brennan DC, Cuburu N, Storch GA, Viscidi RP, Randhawa PS, Buck CB (2012). Neutralization serotyping of BK polyomavirus infection in kidney transplant recipients. PLoS Pathog.

[26] Pastrana DV, Ray U, Magaldi TG, Schowalter RM, Cuburu N, Buck CB (2013). BK polyomavirus genotypes represent distinct serotypes with distinct entry tropism. J Virol.

[27] Randhawa P, Pastrana DV, Zeng G, Huang Y, Shapiro R, Sood P, Puttarajappa C, Berger M, Hariharan S, Buck CB (2015). Commercially available immunoglobulins contain virus neutralizing antibodies against all major genotypes of polyomavirus BK. Am J Transplant.

[28] Schmidt T, Adam C, Hirsch HH, Janssen MW, Wolf M, Dirks J, Kardas P, Ahlenstiel-Grunow T, Pape L, Rohrer T, Fliser D, Sester M, Sester U (2014). BK polyomavirus-specific cellular immune responses are age-dependent and strongly correlate with phases of virus replication. Am J Transplant.

[29] Schmitt C, Raggub L, Linnenweber-Held S, Adams O, Schwarz A, Heim A (2014). Donor origin of BKV replication after kidney transplantation. J Clin Virol.

[30] Schwarz A, Linnenweber-Held S, Heim A, Framke T, Haller H, Schmitt C (2016). Viral origin, clinical course, and renal outcomes in patients with BK virus infection after living-donor renal transplantation. Transplantation.

[31] Solis M, Velay A, Porcher R, Domingo-Calap P, Soulier E, Joly M, Meddeb M, Kack-Kack W, Moulin B, Bahram S, Stoll-Keller F, Barth H, Caillard S, Fafi-Kremer S (2018). Neutralizing antibody-mediated response and risk of BK virus-associated nephropathy. J Am Soc Nephrol.

[32] Thangaraju S, Gill J, Wright A, Dong J, Rose C, Gill J (2016). Risk factors for BK Polyoma virus treatment and Association of Treatment with Kidney Transplant Failure: insights from a paired kidney analysis. Transplantation.

[33] Velay A, Solis M, Benotmane I, Gantner P, Soulier E, Moulin B, Caillard S, Fafi-Kremer S (2019). Intravenous immunoglobulin administration significantly increases BKPyV genotype-specific neutralizing antibody titers in kidney Transplant recipients. Antimicrob Agents Chemother.

[34] Willhelm M, Wilk S, Kaur A, Hirsch HH, Swiss Transplant Cohort S (2019). Can HLA-B51 protect against BKPyV-DNAemia?. Transplantation.

[35] Wunderink HF, De Brouwer CS, Gard L, De Fijter JW, Kroes ACM, Rotmans JI, Feltkamp MCW (2019). Source and relevance of the BK polyomavirus genotype for infection after kidney transplantation. Open Forum Infect Dis.

[36] Wunderink HF, Haasnoot GW, de Brouwer CS, van Zwet EW, Kroes ACM, de Fijter JW, Rotmans JI, Claas FHJ, Feltkamp MCW (2019). Reduced risk of BK polyomavirus infection in HLA-B51-positive kidney Transplant recipients. Transplantation.

[37] Wunderink HF, van der Meijden E, van der Blij-de Brouwer CS, Mallat MJ, Haasnoot GW, van Zwet EW, Claas EC, de Fijter JW, Kroes AC, Arnold F, Touze A, Claas FH, Rotmans JI, Feltkamp MC (2017). Pretransplantation donor-recipient pair Seroreactivity against BK polyomavirus predicts viremia and nephropathy after kidney transplantation. Am J Transplant.

[38] Zhong S, Randhawa PS, Ikegaya H, Chen Q, Zheng HY, Suzuki M, Takeuchi T, Shibuya A, Kitamura T, Yogo Y (2009). Distribution patterns of BK polyomavirus (BKV) subtypes and subgroups in American, European and Asian populations suggest co-migration of BKV and the human race. J Gen Virol.

